# Role of cGMP in fat and metabolism

**DOI:** 10.1186/2050-6511-14-S1-O25

**Published:** 2013-08-29

**Authors:** Alexander Pfeifer, Ana Kilić, Linda Hoffmann

**Affiliations:** 1 Institute of Pharmacology and Toxicology, Sigmund-Freud-Str. 25, D-53105 Bonn, Germany

## Background

The cGMP pathway regulates a large spectrum of physiological processes including metabolism. Our aim is to elucidate the cGMP signaling cascade in adipose tissue. Two types of fat tissue can be distinguished in mammals: white adipose tissue (WAT), which is the biggest storage of energy, and brown fat (BAT) that can dissipate energy as heat (non-shivering thermogenesis). We and others found that cGMP enhances differentiation of brown and white adipocytes [1-4].

## Methods and results

In our studies, we initially focused on protein kinase G (PKG/cGK), which is expressed both in white and brown adipocytes. Using gain- and loss-of-function models, we found that PKG is the major receptor for cGMP in brown adipocytes. We investigated the *downstream* targets of PKG and found a novel negative feedback loop that regulates cGMP levels. Importantly, in the presence of increased cGMP levels, we found an enhanced development of brown-like adipocytes, so-called beige or brite (*br*own in wh*ite*) cells both *in vitro* and *in vivo*. These data indicate that cGMP not only enhances development of “classical” brown adipocytes, but also promotes development of beige cells.

Therefore, we studied the effect of cGMP in white adipocytes in more detail. Lentiviral overexpression of PKG enhanced differentiation of white adipocytes. Moreover, PKG induced the expression of a brown-like adipogenic program in white fat cells. Treatment of mice with the PDE inhibitor sildenafil for only 7 days promoted “browning” of WAT. Further studies regarding the cGMP signaling cascade *upstream* of PKG revealed that both particulate as well as soluble guanylyl cyclases are the source of cGMP in adipocytes.

## Conclusion

In conclusion, cGMP is essential for normal differentiation of white and brown adipocytes. The cGMP/PKG pathway also induces “browning” of white fat and thus could be a promising target for developing novel therapies to treat metabolic diseases that are associated with imbalances in energy homeostasis including obesity and cachexia.
